# Advances in ER-Phagy and Its Diseases Relevance

**DOI:** 10.3390/cells10092328

**Published:** 2021-09-06

**Authors:** Lingang He, Xuehong Qian, Yixian Cui

**Affiliations:** 1 Department of Neurosurgery, Zhongnan Hospital, Wuhan University, Wuhan 430071, China; ysbclgh@whu.edu.cn (L.H.); xuehong_edu@163.com (X.Q.); 2 Frontier Science Center for Immunology and Metabolism, Medical Research Institute, School of Medicine, Wuhan University, Wuhan 430071, China

**Keywords:** ER-phagy, receptor, neurodegenerative diseases, cancer, metabolic diseases

## Abstract

As an important form of selective autophagy in cells, ER-phagy (endoplasmic reticulum-selective autophagy), the autophagic degradation of endoplasmic reticulum (ER), degrades ER membranes and proteins to maintain cellular homeostasis. The relationship between ER-phagy and human diseases, including neurodegenerative disorders, cancer, and other metabolic diseases has been unveiled by extensive research in recent years. Starting with the catabolic process of ER-phagy and key mediators in this pathway, this paper reviews the advances in the mechanism of ER-phagy and its diseases relevance. We hope to provide some enlightenment for further study on ER-phagy and the development of novel therapeutic strategies for related diseases.

## 1. Introduction

Selective autophagy is a class of autophagic pathways that targets specific substrates for degradation. Depending on the substrates, selective autophagy can be classified into mitophagy, lysophagy, aggrephagy, pexophagy, nucleophagy, xenophagy, and ER-phagy (endoplasmic reticulum-selective autophagy), etc. [1]. Receptor proteins determine the selectivity of autophagic substrates.

ER-phagy, which is also called reticulophagy, refers to the selective autophagy of the endoplasmic reticulum (ER). Cells use it to selectively clean up damaged ER subdomains and abnormally accumulated ER luminal proteins to maintain homeostasis. With the discovery of the first ER-phagy receptor in 2015, this field has really entered the study at the molecular level and significant mechanisms have been unveiled. At the same time, a close relationship between ER-phagy and human diseases, such as neurodegenerative diseases, cancers, and metabolic diseases, has also been found. Here, we summarized the current knowledge about ER-phagy and its relationship with human health, hoping to provide some enlightenment for further study.

## 2. The Structure and Function of the ER

ER is a continuous membrane system with lamellar and tubular structures, which is termed sheet ER and tubular ER respectively. As a versatile organelle in eukaryotic cells, it has important functions in numerous cellular processes, such as protein folding and modification, lipid and hormone synthesis, ion storage, metabolism regulation and detoxification [2,3]. According to the attachment with ribosomes, ER can be classified into rough ER and slippery ER. The surface of the rough ER membrane is attached with ribosomes, and it functions in the synthesis of membrane proteins and secreted proteins. In contrast, the surface of the slippery ER membrane is not bound to ribosomes, and it functions in the synthesis of lipids and has physical contact with many other organelles.

When cells are stimulated by external or internal factors which cause a large accumulation of unfolded or misfolded proteins in ER, the homeostasis of ER will be disturbed. To alleviate the ER stress and restore homeostasis, cells activate the self-protection process which is named unfolded protein response (UPR) to upregulate protein folding ability, reduce protein translation, and accelerate ER-associated protein degradation (ERAD) [4,5]. Continuous ER stress and UPR can activate ER-phagy. ER-stress, UPR, and ER-phagy are the main responses of ER, which regulate the dynamic structure and critical functions of ER coordinately.

## 3. ER-Phagy

### 3.1. Definition, Classification, and Function of ER-Phagy

ER is not only a key site for the initiation of macro-autophagy but also a substrate of autophagy. Autophagy with ER as a specific substrate is called ER-phagy or reticulophagy [1]. It occurs both under physiological conditions at the basal level, and when cells are insulted by starvation, UPR, toxin stimulation, and many other internal or external environmental changes, to achieve cell homeostasis by removing damaged or excess ER [1,3,6,7,8].

The phenomenon of ER being engulfed by the double-membrane vesicle, autophagosomes, was first reported in 1973 [9]. Phenobarbital treatment can significantly induce the formation of inner membrane structure within hepatocytes. After removing the drug, the slippery ER is preferentially degraded through the autophagy pathway, which puts forward the concept of ER-phagy [9]. Later, it was found that rapamycin, an mTOR inhibitor that mimics starvation conditions, can also induce the degradation of ER in yeast, which is dependent on the autophagy machinery [10]. Interestingly, typical UPR inducers such as DTT (dithiothreitol, a reducing agent which breaks disulfide bonds within proteins and maintains the sulfhydryl group in a reduced state, causing serve accumulation of unfolded proteins within ER) and thapsigargin (Tg, a sarcoplasmic reticulum/ER Ca^2+^-ATPase inhibitor) induce the formation a special ER structure which is called ER whorl [8,11]. In mammalian cells, Fang Xu et al. found that Tg induces ER whorls, which is dependent on PERK activation and COPII machinery [11]. Sebastián Bernales et al. found that the treatment of yeast cells with DTT leads to the dramatic increase of ER volume and also induces the formation of ER whorls [12]. Late on, the same lab found that these ER whorls can be delivered into the vacuole independent of the core macro-autophagy machinery [8]. Therefore, it was being debated whether vacuolar ER degradation is mediated by the autophagic pathway or not. The big breakthrough of this field happened in 2015 when Keisuke Mochida et al. and Aliaksandr Khaminets et al. reported the first ER-phagy receptors in yeast cells and mammalian cells, respectively [13,14]. The discovery of receptor proteins that target ER into autophagosomes gave a clear answer to the existence of selective autophagic degradation of ER.

Depending on the requirement of the macro-autophagy machinery or not, ER-phagy can be classified into macro-ER-phagy and micro-ER-phagy. Macro-ER-phagy (hereafter ER-phagy) is mediated by ER-resident or ER-associated receptors which recruit autophagy machinery and then target ER into autophagosome [6,7]. Micro-ER-phagy refers to the engulfment of ER by lysosomes directly, and it does not need receptors or the formation of autophagosomes [6,7]. In addition, another form of ER degradation pathway, ER-to-lysosome-associated degradation (ERLAD), has also been reported [15]. It requires ER-phagy receptors but not involves autophagosome formation [15]. This process is highly related to, but distinct from ER-phagy mechanistically.

### 3.2. The Catabolic Process and Regulation Mechanism of ER-Phagy

**Figure 1:**
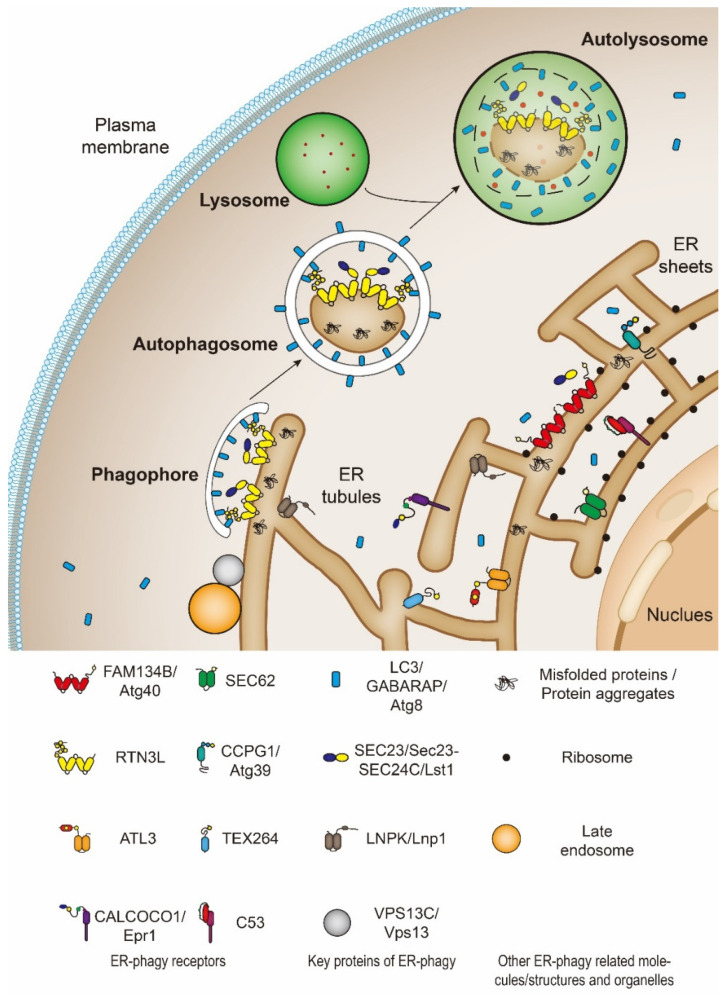
The catabolic process of endoplasmic reticulum-selective autophagy (ER-phagy). ER-phagy receptors concentrate at a specific subdomain of ER (endoplasmic reticulum) which needs to be degraded, being recognized by LC3/GABARAP/Atg8. In this way, ER subdomains are bridged to the autophagic machinery. The isolation membrane linked with ER assembles and expands to phagophore. Then the phagophore wraps the ER fragments and seals to form an autophagosome. Subsequently, the autophagosome fuses with the lysosome to form the autolysosome in mammalian cells (shown here); or the vacuoles in yeast and plants. Eventually, the components engulfed by autophagosomes are degraded by the lysosomal/vacuolar hydrolases. Note: Yeast Atg40, Atg39, and Epr1 are similar to mammalian FAM134B, CCPG1, and CALCOCO1, respectively. These similarities are not only reflected in their structures, but also in their processes of mediating ER-phagy. Thus, the same symbols are used.

ER-phagy is a multistep process that needs specific receptors and core autophagic machinery to promote the degradation of ER components. Under the induction of ER stress, such as UPR, protein aggregation, nutritional deficiency, and the destruction of ER structure, the ER components which need to be degraded can be recognized and “labeled” by specific ER-phagy receptors (Figure 1). At the same time, the cells activate the autophagosome initiation complex mainly by inhibiting mTOR or direct phosphorylation of Atg1/ULK1 (Serine/threonine-protein kinase ULK1, Atg1’s homolog in mammals) by AMPK to initiate the assembly of the isolation membrane. Ubiquitin-like proteins, Atg8/LC3/GABARAP, will be recruited to the growing isolation membrane to help membrane expansion. Meanwhile, Atg8/LC3/GABARAP proteins can identify and directly bind ER-phagy receptors. In this way, the ER subdomains to be degraded as well as the ER-phagy receptors are targeted into the autophagosomes as the isolation membranes gradually grow and seal. After that, the ER-containing autophagosomes fuse with the lysosome to degrade the substrate [2,3]. Regarding the formation of autophagosomes, ER-phagy is very similar to nonselective macro-autophagy. The main difference is that ER-phagy has substrate selectivity and this is achieved through ER-phagy receptors, which will be discussed extensively in Section 4.

Like macro-autophagy, ER-phagy is also regulated by signal transduction [13,14,16]. Different stimuli can induce autophagy in different regions of ER, and the receptors and regulatory factors involved are different. And different receptors may participate in ER-phagy at the same time to preserve cell homeostasis [17]. What’s more, in the process of ER-phagy, the expression level, localization, and post-translational modifications (phosphorylation, ubiquitination, etc.) of receptors and other participating proteins are also tightly regulated. Some post-translational modifications are crucial for ER-phagy [18,19,20,21], which can be seen in another recent review [22].

### 3.3. The Catabolic Process of Micro-ER-Phagy and ERLAD

For micro-ER-phagy: In yeast, when ER-stress appears or ER expands excessively, a part of ER can form a whorled structure. Then it is taken up into the vacuoles by the invaginated vacuolar membrane for degradation [8]. In the mammalian cell, mutated type I procollagen proteins can be recruited to the ERES (ER exit sites) site. COPII coat proteins and autophagy-related proteins (SQSTM1, LC3, etc.) can also be recruited to the same site. Then the ERES that contain these proteins are modified by ubiquitination and engulfed by the lysosomes in a process like micro-autophagy [23].

For ERLAD pathway: When the proteasome-resistant alpha1-antitrypsin Z (ATZ) polymers accumulate in the ER, molecular chaperone proteins such as CALNEXIN can separate the ER subregions where the aggregates locate. Then, with the assistance of ER-phagy receptor—FAM134B, ER-derived, single membrane-bound vesicles are formed in this area. In addition, the association of FAM134B with LC3 can promote the vesicles docking to the membrane of RAB7/LAMP1 positive endolysosomes. Eventually, vesicles containing aggregates are fused with endolysosomes to be degraded [15]. Furthermore, a recent study showed that glucose processing of N-glycans of proteasome-resistant misfolded proteins, such as ATZ, is required for targeting these proteins for degradation by ERLAD [24]. In this process, CALNEXIN works with UDP-glucose: glycoprotein glucosyltransferase (UGGT1) and other glucose processing enzymes to mediate substrates selection [24].

## 4. Key Molecules of ER-Phagy (Receptors, Cofactors, etc.)

Like other selective autophagy, the occurrence of ER-phagy requires specific receptors. In principle, these receptors are either directly or indirectly located on the ER membrane and harbor AIM (Atg8 interaction motif)/LIR (LC3 interaction region)/GIM (GABARAP interaction motif), by which these receptors bind to Atg8/LC3/GABARAP and bridge ER to the autophagosomes.

**Figure 2:**
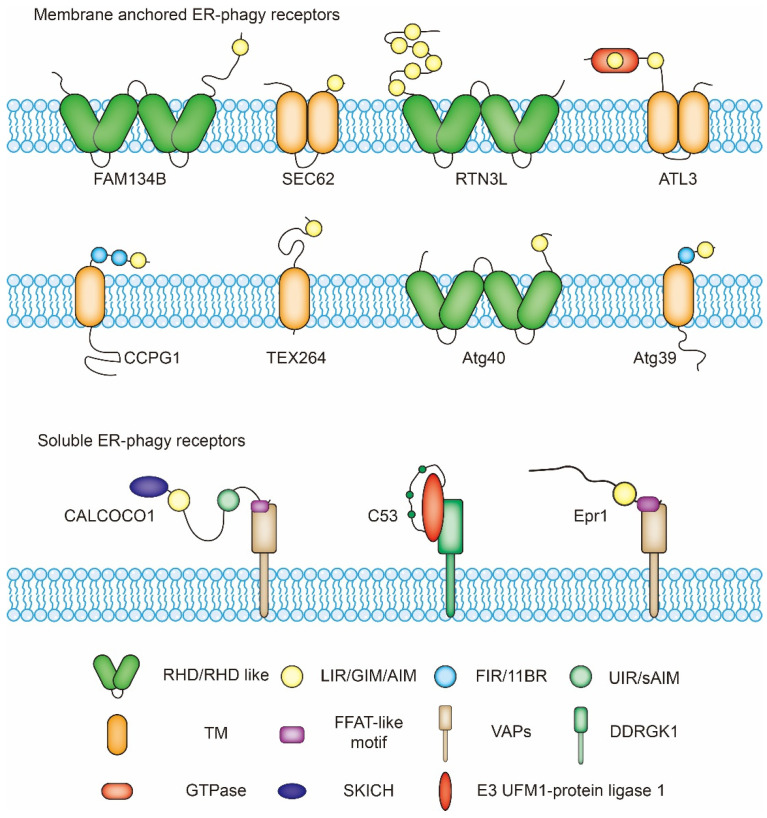
Structure of ER-phagy receptors. FAM134B, SEC62, RTN3L, ATL3, CCPG1, TEX264, and CALCOCO1 are ER-phagy receptors in *Homo sapiens* and other mammals. C53 is the ER-phagy receptor in mammals and plants such as *Arabidopsis thaliana* and *Marchantia polymorpha*. Atg39 and Atg40 are ER-phagy receptors in *Saccharomyces cerevisiae*. Epr1 is the ER-phagy receptor in *Schizosaccharomyces pombe*. RHD, reticulon-homology domain (mammals); RHD-like, putative reticulon-homology domain-like structure (yeast); LIR, LC3-interacting region; GIM, GABARAP-interacting region; AIM, Atg8-interacting motif; FIR, FIP200-interacting region; 11BR, Atg11-binding region; UIR, ubiquitin interacting motif-docking site-interacting region; sAIM, shuffled ATG8 interacting motifs; TM, transmembrane domain; FFAT, two phenylalanines (FF) in an acidic tract (AT); VAPs, vesicle-associated membrane-associated proteins; GTPase, dynamin-like GTPase domain; SKICH, SKIP carboxyl homology domain; DDRGK1, DDRGK domain-containing protein 1.

So far, eleven ER-phagy receptors have been identified from different species. They are either ER-membrane-anchored proteins or ER-membrane-associated proteins (Figure 2). Among them, six in mammals (FAM134B, RTN3L, TEX264, ATL3, SEC62, and CCPG1) and two in budding yeasts (Atg40 and Atg39) are directly anchored on the ER membrane by their reticulon domains or transmembrane domains. The rest identified receptors (CALCOCO1, C53, and Epr1) are located in the ER by binding to ER membrane resident proteins.

In mammalian cells, FAM134B is located on the sheet ER, thus, mediating sheet ER degradation [13]; RTN3L, TEX264, and ATL3 are located on the tubular ER, therefore, responsible for tubular ER clearance [17,25,26,27]; SEC62 is required for recov-ER-phagy, which refers to the ER-phagy induced to restore the homeostasis from ER-stress [28]; CCPG1 involves in maintaining the protein homeostasis of ER [16]; the most-recently discovered ER-phagy receptor, CALCOCO1, is very special because it is the first soluble and non-ER resident protein which mediates the self-bite of ER [29,30]. In budding yeast cells, Atg39 locates on the perinuclear ER (pnER); Atg40 is mainly present on the cytoplasmic ER (cytoER) and cortical ER (cER) [14,31,32]. Epr1 is another soluble ER-phagy receptor protein found in fission yeast and functions like mammalian CALCOCO1 [33,34]. In addition, C53 is a soluble ER-phagy receptor found in both plant and mammalian cells. The discovery of these diverse receptors has greatly promoted the understanding of ER-phagy and enriched the entire field of autophagy. Followed is a concise but comprehensive introduction to each ER-phagy receptor.

### 4.1. Receptors, the Key ER-Phagy Players

#### 4.1.1. FAM134B (Family with Sequence Similarity 134 Member B)

FAM134B is also known as JK-1 or RETREG1 (reticulophagy regulatory factor 1), one of the members of the FAM134 reticulon family. It is the first ER-phagy receptor discovered in mammalian cells and considered as the ortholog of the first identified yeast ER-phagy receptor, ATG40 [14]. FAM134B was identified as a novel LC3B binding protein from a yeast two-hybrid screen and acts as a receptor to mediate the degradation of sheet ER [13]. FAM134B only specifically functions in ER-phagy, as knockout of FAM134B has no obvious effect on other types of selective autophagy or macro-autophagy [13]. The discovery of this first specific receptor enables researchers to confirm that there is selective macro-autophagy for ER, like mitophagy, during which mitochondria are targeted to the autophagosomes for degradation by receptor proteins [35,36].

FAM134B has a reticulon homology domain (RHD) at its N-terminus, which anchors FAM134B to the ER membrane and mediates the remodeling and bending of the ER membrane [37]. Xiao Jiang et al. showed that the RHD of FAM134B can be phosphorylated by CaM kinase II subunit beta (CAMK2B), which consequently promotes FAM134B to form oligomers. The oligomerization of FAM134B is critical for ER fragmentation [38]. The C-terminal of FAM134B has an LIR, which binds the LC3 family proteins LC3/GABARAP [13]. ATL2 (Atalstin2), an ER-resident GTPases, might act downstream of FAM134B to mediate the clearance of damaged sheet ER [39]. In addition, FAM134B can clear misfolded procollagen by ER-phagy with the assistance of CANNEXIN [40]. It also works with CALNEXIN to mediated ATZ degradation by ERLAD [15].

Interestingly, FAM134A and FAM134C, which belong to the same protein family as FAM134B, also bind GABARAP/LC3. FAM134C may be involved in ER-phagy under amino acid starvation. It may also use its LIR to recruit autophagy machinery, and use its RHD to promote the bending and fragmentation of ER [41]. But the involvement of FAM134A in ER-phagy has not been reported yet.

#### 4.1.2. RTN3L (Reticulon-3L)

RTN3 is also known as HAP or NSPLII. RTN3 proteins are enriched in a highly curved ER membrane, especially tubular ER. Among the numerous splice variants, only the isoform of RTN3B, which was renamed RTN3L, functions as the ER-phagy receptor [25]. It can interact with all six ATG8 family members in mammalian cells, but GABARAP-L1 is the preference. RTN3L consists of 1013 AA, which is missing 19 AA compared to the full-length RTN3. The C-terminal has RHD, which anchors RTN3L to the ER and promotes the bending of the membrane. The N-terminal is in the cytoplasm and has 6 LIRs. Only if all the 6 LIRs are mutated can the interaction between RTN3L and LC3/GABARAP be completely abolished. Knockout of RTN3L does not affect other types of selective autophagy or macro-autophagy [25]. In contrast with FAM134B which regulates turnover of ER sheets, RTN3L mediates the selective autophagic degradation of ER tubules. It has been reported that Rtn1/2 may also mediate ER-phagy in maize [42]. These studies suggest that the functions of RTN family proteins in different species are conserved.

The RHD of RTN3L may be able to mediate ER membrane bending and subsequent serving from the network to form fragments during ER-phagy. It has also been reported that the RHD of RTN3L promotes the formation and efflux of Hepatitis C Virus (HCV) exosomes [43]. And LIRs of RTN3L also have other functions besides interacting with LC3/GABARAP. Under non-starvation conditions, RTN3L is recruited to the endosomal MCSs (membrane contact sites) by the binding of its LIR with the FSV domain of Rab9a, regulating endosome maturation and cargo sorting [44].

#### 4.1.3. ATL3 (Atlastin-3)

ATL3 belongs to the dynamin-like GTPase Atlastin family and mediates tubular ER-phagy [27]. ATL3 locates on the tubular ER. It has two transmembrane regions connected by a luminal polypeptide. There is a GTPase domain similar to GTP dynein at its N-terminus, which can undergo trans dimerization with the participation of GTP to mediate tubular ER fusion [45]. ATL3 contains two GIMs, which can bind GABARAP proteins and target tubular ER for lysosomal degradation [27]. Unlike other mammalian receptors, GIMs (core sequences YGRL and KQKL) of ATL3 are not located in essentially disordered regions and partly overlap with the N-terminal dynein-like GTPase domain in the cytoplasm [27,46,47]. Mutation of either GIM will weaken the binding between ATL3 and GABARAP, while the mutation of the GTP binding motif does not affect the interaction of ATL3 with GABARAP or its function in ER-phagy. This observation suggests that its function in catalyzing tubular ER fusion can be separated from its function in ER-phagy [27].

Both ATL3 and RTN3L mediate tubular ER turnover. They may function redundantly. This speculation is supported by the evidence that overexpression of RTN3L can rescue the ER-phagy defect in ATL3-deficient COS-7 cells [27]. In addition, ATL3 and ATL2 interact with ULK1 to promote the recruitment of the ULK1 complex to the ER and the subsequent formation of autophagosomes [46]. This finding implies that ATL3 may have dual functions in ER-phagy. On the one hand, it recruits the ULK1 complex to facilitate phagophore initiation; and on the other hand, it targets ER fragments to the autophagosomes by binding with GABARAP proteins.

As mentioned in Section 4.1.1, ATL2 participates in ER-phagy mediated by FAM134B. After FAM134B binds to the receptor, ATL2 may dimerize itself and promote the bending of the ER membrane by its GTPase activity, thereby assisting the fragmentation of the ER to be degraded [39]. In addition, ATL1, another Atlastin family member which functions in the formation of ER and ER-derived vesicles [48], can interact with GABARAP by its GIM and may also involve in ER-phagy [27]. It is known that ATL3, ATL2, and ATL1 have high sequence similarities [49]. It is speculated that they can functionally compensate each other, such as ER-phagy or tubular ER generation [39,50]. However, some literature suggested that they have some differences in executing functions. For example, the GTPase activity of ATL2 is required for ER-phagy while the GTPase activity of ATL3 might be dispensable for ER-phagy [27,39]. The catalytic activities and dimerization ability of ATL3 are weaker than ATL1/2, which may weaken its ability to mediate morphological changes of ER [51,52]. Thus, when ATL3 acts as an ER-phagy receptor, other machinery may help the bending and fragmentation of ER. Nevertheless, more research is needed to further elucidate their functions in ER-phagy.

#### 4.1.4. SEC62 (Translocation Protein SEC62)

SEC62 is also known as Translocation protein 1. SEC62 and SEC63 are ER transmembrane proteins that bind SEC61, the central translocon component, to mediate the transport of newly-synthesized polypeptides into the rough ER [53,54,55]. Recent studies have found that SEC62 can also act as an ER-phagy receptor in mammalian cells. SEC62 mediates the degradation of excess ER which is formed under stress conditions, such as UPR, to restore ER homeostasis. This process is defined as recov-ER-phagy [28]. In *Arabidopsis*, AtSec62, the homolog of SEC62, is also implicated in ER-phagy under ER stress [56].

SEC62 is located on both sheet and tubular ER and it is a multiple transmembrane protein. The C-terminal cytoplasmic region of SEC62 has an LIR motif, which is essential for ER-phagy but not required for the transport function. Intriguingly, SEC63, which plays a role in SEC62-mediated protein transport, does not affect ER-phagy [28,54]. A possible reason is that SEC62 uses different parts to interact with LC3/GABARAP or SEC63 [57].

It is worth mentioning that autophagosomes formed during SEC62-mediated recov-ER-phagy contain specific UPR-upregulated proteins, including chaperones such as CALNEXIN, but other ER components such as ERAD proteins are largely excluded [28]. This observation once again highlights the selectivity of ER-phagy. However, further study is required to elucidate the mechanism of substrates sorting.

#### 4.1.5. CCPG1 (Cell Cycle Progression Protein 1)

CCPG1 was first discovered to prevent yeast cell cycle arrest [58]. Subsequent studies found that CCPG1 can act as an ER-phagy receptor, mediating ER-phagy in pancreatic cells and other exocrine cells in response to UPR to maintain ER protein homeostasis [16]. CCPG1 localizes to the perinuclear ER and forms small foci at the ER periphery. It has a single transmembrane domain. The C-terminal is inside the ER lumen, and the disordered N-terminal in the cytoplasm has one LIR and two FIRs (FIP200 interacting region), which interact with GABARAP/LC3 and FIP200, respectively, thus linking the ER to the autophagic machinery. The additional binding ability of CCPG1 with FIP200 makes it an uncanonical ER-phagy receptor because most receptors in mammalian cells only bind GABARAP/LC3. During ER stress, the expression level of CCPG1 is upregulated to drive the flux of peripheral ER-phagy. When CCPG1^−/−^ cells were insulted by nutrition deficiency, the degradation of the tubular ER is severely impaired while degradation of sheet ER remains unaffected [16].

#### 4.1.6. TEX264 (Testis-Expressed Protein 264)

Earlier studies have found that TEX264 may be involved in the repair of covalent DNA-protein cross-links during DNA synthesis [59]. In 2019, TEX264 has been identified as a novel ER-phagy receptor by two independent studies [17,26]. TEX264 is a single-pass ER transmembrane protein. The N-terminal of TEX264 is in the ER lumen and the C-terminal is in the cytoplasm which harbors an LIR motif and a long intrinsically disordered region (IDR). LIR and IDR of TEX264 are both indispensable for the function of TEX264 in ER-phagy [26].

Although TEX264 got its name by being identified as a testis-expressing protein, it is indeed ubiquitously expressed in many tissues at a relatively high level, which makes it to be a major ER-phagy receptor [26]. When ER-phagy is induced, TEX264 concentrates and forms a punctate structure in the ER tubule three-way junctions, where LC3 puncta have already localized. According to the live-cell images, it seems likely that the accumulation of TEX264 at ER-phagy sites occurs after LC3 recruitment [17]. This observation seems to contradict the previous speculation that ER-resident receptors may concentrate firstly at specific ER subdomains and serve as an “eat me” signal and then trigger the initiation of ER-phagy by recruiting LC3 proteins. In the case of TEX264-mediated ER-phagy, it is very puzzling how LC3 recognizes specific ER subdomains before the receptor proteins concentrate.

#### 4.1.7. CALCOCO1 (Calcium-Binding and Coiled-Coil Domain-Containing Protein 1)

CALCOCO1 is the most recently discovered ER-phagy receptor [29,30]. Different from other receptors, CALCOCO1 is a soluble protein that does not have a transmembrane domain or reticulon domain to facilitate its location on the ER membrane. Instead, CALCOCO1 localizes to the ER by interacting with VAPA and VAPB, two VAMP-associated, ER-membrane-localized proteins via its FFAT-like motif [29].

Thaddaeus Mutugi Nthiga et al. found that the N-terminal of CALCOCO1 has an atypical LIR core motif (LVV), which mediates ER-phagy through binding with ATG8 family proteins (especially GABARAP subfamily) [29]. It does not have a UIM (ubiquitin interacting motif) like motif, which can bind UDS (ubiquitin docking site) of ATG8 family members. However, the C-terminal of CALCOCO1 has a motif called UIR (UDS interacting region), which can bind UDS and assist LVV to bind ATG8 family members.

Depletion of CALCOCO1 leads to inefficient basal autophagy flux and ER expansion [29,30]. However, another article found that CALCOCO1 has a traditional LIR in its SKICH (SKIP carboxyl homology) domain and mediates ER-phagy by this LIR and CLIR (a non-canonical LC3C-interacting region) [30]. And in this article, the preferred ATG8 family proteins of CALCOCO1 are LC3C, LC3B, and GABARAPL2 [30]. Therefore, the specific mechanism of ER-phagy mediated by CALCOCO1 remains to be further studied.

Additionally, it has been reported that CALCOCO1 may interact with ZDHHC17 (Golgi resident protein) through its C-terminal ZDHHC-AR-binding motif to mediate the autophagic degradation of Golgi [60]. CALCOCO1 has also been found to function in other selective autophagy such as submitochondrial mitophagy and ferritinophagy [30].

#### 4.1.8. Atg40 (Autophagy-Related Protein 40)

As mentioned earlier, Atg40 is the ortholog of mammalian ER-phagy receptor FAM134B. Atg40 locates in cER and cytoER and is responsible for their degradation in *Saccharomyces cerevisiae*. The ER-phagy is largely blocked when *ATG40* is knocked out, and almost completely suppressed when *ATG39* is deleted simultaneously [14].

The RHD at the N-terminal of Atg40 can induce ER membrane bending and fragmentation, and the AIM at the C-terminal can bind Atg8. If either of them is missing, Atg40′s function in ER-phagy will be disrupted. Atg40 is recruited to ER-isolation membrane contacts, where it interacts with Atg8, forms oligomers, and promotes the fragmentation of ER [38,61]. Similarly, when Atg40 mediates ER-phagy, it requires the core mechanism of macro-autophagy, and the assistance of Lst1 and Lnp1 which will be described below [14,61,62,63].

#### 4.1.9. Atg39 (Autophagy-Related Protein 39)

Atg39 was identified as the receptor of perinuclear ER-phagy in *Saccharomyces cerevisiae* [14]. Atg39 locates in the pnER, which is considered as part of the nuclear envelope. It contains a transmembrane domain, and its N-terminus is in the cytoplasm with an AIM [14]. Atg39 and Atg40 are both implicated in interacting with Atg11, an adaptor that recruits the autophagosome machinery by forming an adaptor-receptor-cargo complex [64,65]. However, only Atg39 has been shown to contain the Atg11 binding region (11BR) [14]. Further study is needed to unveil the 11BR of Atg40. The way which Atg39 functions as a receptor is similar to mammalian CCPG1, which also binds to LC3 and FIP200 (a putative functional homolog of Atg11) simultaneously [16]. Besides mediating perinuclear ER degradation, it was found that some of the nucleus material is also degraded by Atg39-mediated autophagy [14]. Thereby, Atg39 is also considered a nucleophagy receptor [32,66,67]. 

#### 4.1.10. Epr1(ER-Phagy Receptor 1)

Epr1 can mediate ER-phagy induced by DTT, and it is so far the only known ER-phagy receptor in fission yeast, *Schizosaccharomyces pombe* [34]. Like mammalian CALCOCO1, Epr1 is also a soluble protein and is located in the ER by binding with ER-resident VAP proteins, Scs2 and Scs22, via its FFAT domain. When ER-phagy occurs, Atg8 proteins are recruited to the ER by Epr1′s AIM. Both AIM and FFAT are in the C-terminal IDR of Epr1 and indispensable for efficient ER-phagy [34]. And Epr1 can be up-regulated by Ire1 upon DTT treatment [34]. Interestingly, ER-phagy induced by nitrogen starvation in fission yeast is not mediated by Epr1 [34], indicating that other receptors are participating.

#### 4.1.11. C53 (CDK5 Regulatory Subunit-Associated Protein 3)

The latest research shows that C53 is a conserved ER-phagy receptor in both plant and mammalian cells. In normal conditions, C53 localizes in the cytoplasm and nucleus [68,69]. When ER stress increases, C53 will be recruited to ER through transport vesicles [69]. Then together with UFMylation E3 ligase UFL1 and DDRGK1 (UFL1′s membrane adaptor, which anchors in ER membrane), C53 forms a receptor complex to mediate ER-phagy [69]. Unlike most ER-phagy receptors, C53 interacts with ATG8 family proteins through non-canonical shuffled ATG8 interacting motifs (sAIM), which is in the IDR of C53 [69]. And ER-phagy mediated by C53 does not respond to conventional ER-stress induced by carbon or nitrogen starvation, or UPR, but responds to phosphate starvation and ribosome stalling [69]. Like CCPG1, C53 can also interact with FIP200 (mammalian) or ATG11 (*Arabidopsis* *thaliana*) [69].

### 4.2. Other Important ER-Phagy Players

#### 4.2.1. SQSTM1 (Sequestosome-1)

SQSTM1, also named p62, is a well-characterized macro-autophagy adaptor. It has both ubiquitin-associated (UBA) domain and LIR, which enable its binding with polyubiquitylated substrates and LC3 decorated autophagosomes respectively. Recently, it was reported that SQSTM1 also involves in ER-phagy [20]. SQSTM1 can bind to the ubiquitylated E3 ligase TRIM13 which locates on the ER membrane to mediate ER-phagy [20]. In this process, N-terminal arginylation of proteins, such as BIP, can induce SQSTM1 oligomerization, which is essential for ER-phagy mediated by SQSTM1 and TRIM13 [20]. This pathway is responsible for the disposal of misfolded ER protein aggregates which are difficult to be degraded by the proteasome, such as the aggregates of ATZ [20]. Mouse liver cells lacking SQSTM1 fail to degrade excess ER, indicating the important role of SQSTM1 in ER clearance [19].

#### 4.2.2. SEC24C (Protein Transport Protein SEC24C)/Lst1 (Lethal with Sec13 Protein 1)

SEC24C is a cargo adaptor that binds with SEC23 and forms the inner shell of COPII vesicle which mediates ER-to-Golgi traffic. There are four SEC24 proteins in mammals, SEC24A, B, C, and D; three in yeast, Sec24, Iss1, and Lst1. Yeast Lst1 is considered the closest ortholog of mammalian SEC24C. Yixian Cui et al. recently found that Lst1/SEC24C assists receptor proteins, Atg40/FAM134B/RTN3L, to mediate ER-phagy [62,63]. Lst1 binds to Atg40 and promotes the packaging of ER fragments into autophagosomes in *Saccharomyces cerevisiae*. In mammalian cells, SEC24C is required for the lysosomal degradation of FAM134B and RTN3L. And SEC24C/Lst1 themselves can be transported to lysosomes along with autophagosomes when mediating ER-phagy. Lst1/SEC24C has another important role in ER-phagy. It combines with Sec23/SEC23 to form ER-phagy sites (ERPHS), which is distinct from specialized ERES where COPII vesicles on the secretory pathway bud and transport to the Golgi. Lst1 involves both starvation-induced and protein aggregates accumulation-induced ER-phagy [62].

#### 4.2.3. Lnp1 (Lunapark Family Member1)

Yeast Lnp1 is an ER membrane protein which locates at the three-way junction of ER [70]. It functions in the formation and remodeling of the dynamic ER network by interacting with reticulum proteins, Rtn1/Yop1 and Sey1 (the homolog of mammalian Atlastins in yeast). Deletion of Lnp1 results in collapsed and densely reticulated ER [70]. It was found recently that Lnp1 also involves in Atg40-mediated-ER-phagy. Lnp1 is required for the recruitment of Atg40 to the ER-phagy sites and its co-localization with Atg11. Consequently, *lnp1* mutant displays decreased packaging of ER into the autophagosomes [62,71].

Regarding the function as an ER remodeler, the mammalian ortholog of Lnp1, LNPK, also plays a similar role in regulating ER dynamics [39,45]. LNPK enhances the stability of the tubular ER connection point. The lack of LNPK disrupts the formation of the tubular ER network and results in a flatter sheet-like, abnormal ER structure [72]. However, it is unclear whether LNPK plays a similar role in mammalian ER-phagy or not.

#### 4.2.4. Vps13 (Vacuolar Protein Sorting-Associated Protein 13)

Yeast Vps13 locates at numerous membrane contact sites and functions in vacuolar protein sorting. There are four Vps13 homologs in mammalian cells, VPS13A, B, C, and D, among which VPS13A/C has the highest similarity to yeast Vps13 [73]. Like Lst1 and Lnp1, researchers have found that yeast Vps13 functions together with Atg40 in cortical ER-phagy. Vps13 is responsible for assisting the ER fragments to be targeted into the autophagosomes, as its deletion decreases the number of ER-containing-autophagosomes. Yeast Vps13 has a lipid transport function [74]. Therefore, Vps13 may help autophagosomes to encapsulate ER components to be degraded by promoting lipid transport. However, further research is still required to test this possibility, as well as dissect the exact role of Vps13 and its mammalian orthologs in ER-phagy.

### 4.3. Transcription Factors in ER-Phagy

Since excessive or insufficient ER-phagy will affect cell homeostasis, cells use a variety of mechanisms to tightly control ER-phagy, including regulation of ER-phagy players’ expression at different levels. And transcription factor is one of the main elements regulating the expression level of proteins. Therefore, understanding the influence of these elements on ER-phagy may help us to deeply explore the regulatory mechanism of ER-phagy.

#### 4.3.1. TFEB and TFE3 (Transcription Factor EB/E3)

TFEB and TFE3 are master transcriptional regulators of autophagy [75]. They can be activated by nutrition insufficiency and promote autophagic flux by upregulating the transcription of genes responsible for the biogenesis of autophagosomes and lysosomes. A recent study found that fibroblast growth factor 18 can promote the nuclear translocation of TFEB and TFE3, thereby enhancing the transcription of FAM134B and subsequent ER-phagy in chondrocytes [76]. More importantly, this process is physiologically relevant to the skeletal development in both fish and mice.

#### 4.3.2. C/EBPβ (CCAAT/Enhancer-Binding Protein β)

C/EBPβ is a transcription factor that is involved in the immune response and autophagy [1,77,78]. A recent study showed that when the mice have fasted, C/EBPβ can bind *FAM134B* promoter and promote expressing of FAM134B-2, a truncated isoform of FAM134B which possesses LIR and is expressed specifically in the liver [79]. FAM134B-2 may promote the degradation of secreted protein apolipoprotein C-III retained in ER via lysosomes [79]. However, it cannot mediate the degradation of bulk ER [79]. Therefore, the role of C/EBPβ regulated FAM134B-2-mediated-ER-phagy remains to be further explored.

#### 4.3.3. Rpd3L (Histone Deacetylase Large Complex)

Rpd3L can suppress the transcription of multiple autophagy genes, including Atg8/LC3 and Atg9 [80,81]. The expression of yeast Atg40 responds to ER stress [14,62]. Yixian Cui et al. found that the upregulation of Atg40 requires the deactivation of Rpd3L [62]. Loss of Pho23 and Rpd3, two components of Rpd3L [82], leads to increased mRNA and protein level of Atg40, but not the target of UPR such as Lst1 or Kar2 [62]. This suggests that the transcriptional repressor of core autophagy machinery may also suppress the transcription of ER-phagy receptors such as Atg40.

#### 4.3.4. Mig1 and Mig2 (Regulatory Protein Mig 1 and 2)

Atg39, another ER-phagy receptor in *Saccharomyces cerevisiae*, is negatively regulated by transcriptional factors Mig1 and Mig2 [83]. Mig1/2 can bind the promoter of *ATG39* and repress its transcription under normal conditions [83]. When ER stress occurs, Snf1 phosphorylates Mig1/2 and promotes their nuclear export [83]. Thus, the suppression of *ATG39* by Mig1/2 is removed to promote the occurrence of ER-phagy [83].

To summarize Section 4, multiple ER-phagy receptors have been identified and characterized. They have different tissue-specific expression patterns, located in different areas of ER, and respond to different biological and environmental cues. These receptors can either function cooperatively or independently to mediate ER-phagy to maintain or restore ER homeostasis. For instance, SEC62, a ubiquitously expressed protein, participates in “recov-ER-phagy” after ER-stress is resolved [28]. CCPG1 appears to be specifically essential in gastric chief cells and pancreatic cells [16]. It is activated when secretory ER stress occurs [16]. In addition, ER-phagy receptors interact with other assistant proteins to determine cargo selectivity. For example, FAM134B forms a complex with CALNEXIN to clear misfolded procollagen or ATZ, a proteasome resistant aggregates [15,40]. ER-phagy receptors may also be linked to components of the COPII complex to promote the sprouting and rupture of the ER by autophagosomes [62]. However, a lot of critical questions about ER-phagy receptors still need to be addressed, such as the role of receptor oligomerization, transcriptional regulation of receptors, and post-translational modification of receptors.

Although a general picture of ER-phagy is established, important issues remain unresolved. A central question is how ER fragmentation and autophagosomes formation are coordinated? Current research shows that there are two possible working models: (1) The fragments of the ER that contains unfolded proteins or protein aggregates are firstly separated from the ER, and then recognized and swallowed by the autophagosomes. (2) Autophagosomes are formed at the proximity of a specific ER subdomain and then wrap the subdomain of ER to be degraded. Further investigation is needed to address which one of the models is correct.

## 5. The Diseases Relevance of ER-Phagy

With the deepening of research on ER-phagy, the association between ER-phagy and human diseases has been gradually revealed. As a crucial basic biological process, ER-phagy requires to be highly controlled, and insufficient or overactivated ER-phagy could be both problematic. The human diseases which have been related to ER-phagy are summarized below based on the type of diseases.

### 5.1. Neurodegenerative Diseases

Neurodegenerative disorders can be characterized by incorrect folding of certain proteins or changes in the intracellular stress response [84]. As an important way to maintain the homeostasis of ER, the main site of protein folding and modification in a cell, ER-phagy plays an important role in clearing misfolded proteins and dealing with intracellular stress. Studies have shown that dysregulated ER-phagy causes a variety of neurodegenerative diseases.

#### 5.1.1. FAM134B and Hereditary Sensory and Autonomic Neuropathy (HSAN)

The mutations of the ER-phagy receptor, FAM134B, have been linked to HSAN type II disease. Different mutations in FAM134B coding sequence which lead to decreased or increased ER-phagy activities have both been linked to HSAN II. For instance, some mutations in *FAM134B* lead to the generation of truncated proteins that lack LIR or LIR plus a part of RHD, such as c.433 C > T (p.Q145X), c.926 C > G (p.S309X). Accordingly, they cannot bind LC3/GABARAP family members or mediate ER-phagy. In this case, impaired ER-phagy flux may contribute to the pathogenesis of HSAN II [85,86,87,88]. However, other mutations identified from HSAN II patients, such as c.646 G > A (p.G216R), may make FAM134B overactive, thus inducing excessive ER-phagy. In this case, too much ER-phagy seems to be responsible for the death of the sensory neurons [2,38,85].

#### 5.1.2. FAM134B and Niemann-Pick Type C Disease

FAM134B-mediated ER-phagy has also been associated with Niemann-Pick type C disease, a progressive and fatal neurodegenerative disease. This disease is caused by mutations in the genes encoding NPC1 or NPC2 proteins [89]. Studies have shown that the majority of the I1061T mutant of NPC1, the most common disease-causing mutant, can be degraded by FAM134B-mediated ER-phagy [90]. A few can be degraded by proteasomes through the MARCH6-dependent ERAD pathway [90]. This implicates that upregulation of ER-phagy and ERAD could be both beneficial for Niemann-Pick type C disease patients from the aspect of eliminating these detrimental mutant NPC proteins.

#### 5.1.3. ATL3/ATL1 and Neurodegenerative Diseases

Mutations in ATL3 and ATL1 have been linked to neurodegenerative diseases, which may be caused by impaired ER-phagy. For example, ATL3 Y192C [91] (located in the first GIM region) mutation and P338R [92] mutation, which were found in HSAN type I patients, can weaken the interaction between ATL3 and GABARAP. This decreased interaction prevents ATL3 from promoting ER-phagy [27]. And Laura Behrendt et al. also showed that Y192C mutation of ATL3 results in impaired autophagy, abnormal ER morphology, and neuronal axon growth defects [93].

Mutations identified in ATL1 have been linked to autosomal dominant hereditary spastic paraplegia (HSP) [94]. The Y196C mutation of ATL1 found in HSP patients [95] also weakens the interaction of ATL1 with GABARAP [27]. Like ATL3 Y192C, this mutation may affect the occurrence of ER-phagy in the central nervous system where ATL1 is ubiquitously expressed [27]. Thus, it is reasonable to speculate that the mutations of ATL1 and the consequent ER-phagy defect contribute to the pathogenesis of HSP.

#### 5.1.4. RTN3 and Alzheimer Disease (AD)

RTN3L-mediated ER-phagy may affect the progression of Alzheimer’s disease (AD). Systematic analyses of RTN3 mutations in AD patients have identified multiple potentially causative mutations [96]. And RTN3 is found to interact with β-secretase 1 (BACE1), which cleaves amyloid precursor protein (APP) and generates amyloid β-protein (Aβ), the major protein existing in Alzheimer’s plaques [97,98]. RTN3 null mouse displayed increased BACE1 protein level and Aβ deposition [98], which could be a consequence of impaired ER-phagy. Moreover, mutated APP could accumulate in the ER and requires the degradation by ER-phagy. Although the mechanism is still unclear, it appears that the pathogenesis of AD is highly relevant to ER-phagy.

#### 5.1.5. VPS13C and Parkinson Disease (PD)

VPS13C mutations have been associated with early-onset PD. In the mammalian cell, VPS13C can be located at the ER-late endosomal contact sites [74], and the outer membrane of mitochondria [99]. Studies in the cell model have shown that the silencing of VPS13C leads to mitochondrial damage as well as abnormally elevated mitophagy [99]. Interestingly, deletion of Vps13, the homolog of VPS13C in yeast, leads to profoundly decreased ER-phagy activity [73]. Therefore, it is quite reasonable to speculate that VPS13C-mediated ER-phagy may also influence the progress of PD.

### 5.2. Cancers

The role of ER-phagy in tumorigenesis is complex. On the one hand, ER-phagy can attenuate excessive ER stress and aid the proliferation and survival of cancer cells. On the other hand, ER-phagy may also induce the death of cancer cells. Its function in tumorigenesis is highly contextual, and likely dependent on the cancer type, progression stage as well as microenvironments.

#### 5.2.1. FAM134B

The ER-phagy mediated by FAM134B can promote cancer survival. Reducing phospholipid biosynthesis via ER-phagy mediated by FAM134B is critical for the proliferation and clonogenicity of mutant isocitrate dehydrogenase 1 (IDHmut) gliomas [100]. This is evidenced by the fact that the blocking of ER-phagy via silencing FAM134B or using autophagy inhibitors (such as bafilomycin A1 and chloroquine) suppresses cancer growth, and increases the lifespan of mice bearing orthotopic IDHmut glioma [100]. Another study in colorectal carcinoma found that FAM134B-mediated ER-phagy can relieve the UPR induced by the therapeutic drug— brigatinib, and then promote the survival of cancer cells. Inhibition of FAM134B-mediated ER-phagy increases the sensitivity of colorectal carcinoma to brigatinib [101].

On the contrary, superabundant ER-phagy mediated by FAM134B induces autophagic cell death of cancer cells. A small chemical, Z36, has been reported to induce FAM134B expression in HeLa cells and promote excessive ER-phagy, and eventually cause cancer cell death [102]. In addition, FAM134B has been reported to suppress the growth of colorectal carcinoma and breast carcinoma, independent of ER-phagy [103,104,105]. Further study is needed to explore whether FAM134B-mediated ER-phagy has a killing effect on cancer in clinical practice.

#### 5.2.2. SEC62

Studies have found that in non-small cell lung cancer (NSCLC) and thyroid cancer, the high expression of SEC62 can make cancer cells more resistant to UPR and other ER-stress, and thus promote the invasion and migration of cancer cells [106]. After inhibiting the expression of SEC62, NSCLC, prostate cancer, and thyroid cancer cells are more sensitive to ER stress-induced by thapsigargin [106,107]. Likewise, silencing of SEC62 in HeLa cells also inhibits their migration [108]. These combined studies suggest that the recov-ER-phagy mediated by SEC62 may help cancer cells to better cope with ER-stress, and promote their survival and migration.

#### 5.2.3. CALCOCO1

N-terminal R12H of CALCOCO1 was found to be one of the risk factors of breast cancer in previous studies [109]. Like the point mutation in CLIR of CALCOCO1, the N-terminal R12H mutation of CALCOCO1 also weakens the binding between CALCOCO1 and LC3C to a similar degree [30]. Therefore, it seems likely that the impaired ER-phagy caused by CALCOCO1 mutation could contribute to the occurrence of breast cancer.

#### 5.2.4. C53

C53 can inhibit cancer cell proliferation by regulating p53 and CDK1-cyclin B1 [68,110]. However, previous reports have also shown that C53 is highly expressed in hepatocellular carcinoma (HCC) cell lines and may be involved in promoting cancer invasion and metastasis [111]. C53-mediated ER-phagy can respond to ER stress caused by blocked protein translation. And cancer cells have very active protein synthesis and thus ER is always stressed. Therefore, it can be speculated that C53, like SEC62, may help cancer cells cope with ER stress and promote their survival through upregulating ER-phagy.

### 5.3. Metabolic Diseases

To maintain proteostasis, multiple degradation pathways, including proteasomal and lysosomal degradation pathways, work together to clear aberrant protein in cells. ER-phagy can target misfolded proteins accumulated in ER to the lysosomes for degradation. When ER-phagy is deficient chronically, it may cause metabolic diseases. In addition, metabolic diseases are not only associated with macro-ER-phagy but also associated with micro-ER-phagy and ERLAD pathways.

#### 5.3.1. Diseases Caused by Misfolded Procollagen

Collagen is the most abundant protein in mammals. The synthesis of collagen is error-prone as it is a huge protein and difficult to be folded correctly. Inefficient secretion of collagen is associated with many human diseases such as lysosomal storage disease or osteogenesis imperfect. Cells deploy multiple strategies to clear misfolded procollagens to control the quality of this protein. Yoshihito Ishida et al. found that misfolded procollagen monomer is cleared via ERAD, while aggregated procollagen is degraded via autophagy [112]. Later, a study by Alison Forrester et al. showed that CALNEXIN assists FAM134B to mediate the degradation of misfolded procollagen via ER-phagy. When FAM134B or CALNEXIN is knocked out, misfolded procollagens accumulate, ER stress increases, and eventually, cell death happens [40,113]. In addition, some type I procollagen mutant proteins are directly transported to the lysosomes for degradation through ERES. In this process, misfolded type I procollagens, COPII envelope proteins, and autophagy-related proteins (SQSTM1, LC3, etc.) are recruited to ERES. Then these ERES containing misfolded type I procollagens are modified by ubiquitination. At last, they are directly engulfed by nearby lysosomes in a process similar to micro-autophagy [23].

#### 5.3.2. Liver and Lung Diseases Associated with α1-Antitrypsin Deficiency (ATD)

The Z variant of human α-1-antitrypsin (ATZ) is the most prevalent cause of ATD related lung and liver disease. ATZ is prone to be misfolded and accumulated as aggregates in the ER, which are difficult to be degraded by the ERAD pathway. Then these aggregates induce UPR, ER damage, and also cause ATD, which further aggravates liver cell apoptosis and injury [20]. When ATZ is overexpressed in yeast, excess ATZ is delivered into the vacuole through vacuolar protein sorting or autophagy [114]. Yixian Cui et al. have found that the later pathway is actually Atg40-Lst1-mediated ER-phagy [62]. In the mammalian cell, SQSTM1 and TRIM13 can bind to ATZ aggregates and make them degraded by ER-phagy [20]. In this way, ER stress caused by ATZ aggregation can be reduced. Then it protects liver cells, reduces liver damage and the potential risk of liver cancer. In addition, ATZ aggregates can also be eliminated through the FAM134B and CALNEXIN-mediated ERLAD pathway [15]. This suggests that the ERLAD pathway may complement ER-phagy.

#### 5.3.3. Diseases Caused by Misfolded Hormone Precursor Proteins

Hormones are synthesized in the ER as precursors and are then processed into their mature form, and secreted out of the cell. Hormones function as important signal molecules regulating the physiology and behavior of multicellular organisms. Misfolded hormone precursor proteins accumulated in the ER can be cleared by ER-phagy. RTN3L-mediated ER-phagy has been reported to broadly degrade these proteins, such as *Akita* proinsulin, proopiomelanocortin C28F mutant (POMC C28F), pro-arginine-vasopressin G57S mutant (pro-AVP G57S), etc. [115]. *Akita* proinsulin is the proinsulin C96Y mutant, one of the causes of mutant *INS*-gene-induced diabetes of youth (MIDY). The soluble monomer form of *Akita* proinsulin can be degraded by ERAD with the help of chaperone GRP170. However, *Akita* proinsulin can also form large aggregates and interferes with the wild-type proinsulin, resulting in insufficient insulin secretion. More harmfully, aggregated *Akita* and wild-type proinsulin cause ER-stress and ER damage, which could further induce islet β cells apoptosis. ER-phagy mediated by RTN3L could effectively remove these aggregates and maintain the soluble state of normal proinsulin for subsequent processing and secretion. Thus, upregulation of ER-phagy mediated by RTN3L could protect islet β cells and alleviate the symptoms associated with MIDY [115,116]. The promotion of RTN3L-mediated ER-phagy by small molecule drugs could be a potential treatment for MIDY, as well as other complications caused by misfolded hormone precursor proteins.

### 5.4. Viral Infection and Innate Immunity

Viruses invade host cells through a variety of methods to avoid cell detection and immune response. To get the materials, machinery, and location for their replication, viruses hijack cellular organelles. ER is utilized by various viruses. For instance, β-coronavirus can hijack ER chaperone protein GRP78/BIP and CALRETICULIN to assemble themselves [117]. Afterward, they transport into the lysosomes and disrupt their function by suppressing lysosomal pH decline. At last, matured β-coronavirus successfully transport out to infect other cells [117]. To protect from viral infection, cells have evolved multiple strategies, including ER-phagy. However, in most cases, viruses actively impede ER-phagy and other immune responses of cells to fulfill their proliferative needs. In these processes, pathogens usually impair the normal function of ER-phagy receptors and ER-phagy related molecules, then hijack them. The next part will introduce the possible relationship between ER-phagy, virus invasion, and innate immunity.

#### 5.4.1. Antiviral Role of FAM134B-Dependent ER-Phagy

FAM134B-dependent ER-phagy may play a role in the response to the Ebola virus (EBOV) and flaviviruses such as Dengue and Zika viruses. Specifically, it can inhibit the replication of the Ebola virus in MEF cells [118]. And in human microvascular endothelial cells, FAM134B-mediated ER-phagy can also degrade the excess ER induced by flaviviruses and the viral proteins encapsulated in ER [119]. This process reduces the supply of vesicle membrane, which is needed for the budding of the virus, and thus restricts viral replication. To fight against the restriction, the viruses use their protease complex, NS2B3, to cleave FAM134B at a site within its RHD. Consequently, FAM134B is unable to oligomerize and mediate ER-phagy to limit the replication of flaviviruses. These findings suggest that developing drugs which target NS2B3 and maintain the normal flow of FAM134B-mediated ER-phagy may help the treatment of flaviviruses [2,119].

#### 5.4.2. Divergent Roles of Atlastins in Flavivirus Infection

Atlastin family proteins have been shown to be involved in the replication and assembly of flaviviruses differently. ATL2 plays a role in the fitting of vesicles for viral replication on ER, while ATL3 is involved in the assembly and transport of mature virions together with ARF4/5 [120]. Another study found that ATL3 is recruited to the viral replication site and interacts with NS2A and NS2B3 of the Zika virus [121]. These studies provide evidence supporting that Atlastins are a central hub for viral replication and maturation. However, it is unclear whether Atlastins mediated ER-phagy plays a role in viral infection. Therefore, it is worthwhile to further investigate how Atlastins are hijacked by viruses and their function as ER-phagy players in antiviral infection, as well as the underlying mechanisms.

#### 5.4.3. RTN3/RTN4 and Viral Infection

RTN3 and RTN4 have been shown to support Simian virus 40 (SV40) infection. RTN3 (including RTN3L) and RTN4 are recruited to the viral foci on the ER, where SV40 emerges and escapes to the cytoplasm [116]. The membrane bending ability of RTNs presumably provides the flexibility of ER membrane to facilitate the viral escape [116,122]. Similarly, RTN3L can induce the bending of the ER membrane and promote the formation and excretion of exosomes that contain HCV [43]. Under viral infection conditions, RTNs are hijacked by the virus to help their escape and the physiological functions of RTNs in ER-phagy may be impaired. Apart from these findings discussed above, the role of RTNs mediated ER-phagy in viral infections is still unclear.

#### 5.4.4. ER-Phagy and Innate Immunity

Studies have shown that ER-phagy participates in the innate immune response. In this process, Cyclic di-adenosine monophosphate (c-di-AMP) of Gram-positive bacteria can be detected by MITA (Mediator of IRF3 activation)/STING (Stimulator of interferon genes protein) in cells. And then, c-di-AMP induces ER-stress and ER-phagy. However, it is unknown which ER-phagy receptor is involved in this process [123]. Subsequent studies have shown that cGAMP induces the interaction of SEC24C with MITA/STING [124]. And with the help of WIPI2, LC3, and SEC24C, MITA/STING mediates non-classical autophagy to remove viral DNA at ERGIC (ER-Golgi intermediate compartment) [124].

The above processes do not conflict with another well-known pathway, in which cGAS-cGAMP induces interferon production by binding to the C-terminal of MITA/STING. These pathways can involve the initiating of innate immune responses against DNA virus infection [124,125]. In addition, studies have shown that SEC24C may assist RTN3L in ER-phagy [62]. Therefore, RTN3L is a possible receptor to mediate ER-phagy to help fight against Gram-positive bacteria invasion. And RTN3L may also mediate ER-phagy to assist the innate immune signal transduction of MITA/STING. But further explorations are needed to dissect the function of ER-phagy in innate immunity.

### 5.5. Other Diseases

#### 5.5.1. CCPG1-Mediated ER-Phagy and Pancreatic Diseases

Upon ER stress, cells increase the synthesis of CCPG1 to promote ER-phagy to attenuate the stress [16]. Insufficient CCPG1 causes the accumulation of protein aggregates and the loss of proteostasis. In an animal model, deletion of CCPG1 results in the loss of the polarity of mouse exocrine cells such as pancreatic cells, and also makes aging mice more sensitive to inflammation [16]. These findings suggest that impaired ER-phagy may be related to disorders of the pancreas such as pancreatitis and pancreatic cancer [2].

#### 5.5.2. TEX264 and Diseases

Although it has not been reported that TEX264-mediated ER-phagy is directly associated with certain diseases, TEX264 should have close disease relevance regarding its function as a major ER-phagy receptor. Research has shown that the expression level of TEX264 is decreased in clear cell renal cell carcinomas [126]. Other studies have shown that TEX264, together with p97 ATPase and SPRTN metalloproteinase, participates in the repair of Topoisomerase 1 cleavage complex (TOP1CC). TOP1CC belongs to covalent DNA-protein crosslinks (DPCs, a large complex that can obstruct chromatin-based processes) and is highly toxic to cells [59,127]. In this process, TEX264 can recognize SUMO-mediated topoisomerase 1(TOP1) and interact with p97 [59]. Interestingly, Cdc48 (p97 homolog), the SUMOylation system, and autophagy are also involved in repairing DPCs in yeast [128]. Therefore, TEX264 may also repair TOP1CC and other DPCs through ER-phagy. In addition, TOP1CC is believed to be involved in neurological diseases and is one of the killing mechanisms of some anti-cancer drugs, such as camptothecin. Thus, ER-phagy mediated by TEX264 may involve in the above diseases by affecting TOP1CC.

#### 5.5.3. C53 and Development

Recent studies have found that C53 is critical in maintaining intestinal development and homeostasis. Intestinal epithelial cell-specific deletion of C53 leads to the absence of Paneth cells and increased susceptibility to experimentally induced colitis [129]. A possible reason for these phenomena is that C53-mediated-UFMylation-dependent-ER-phagy is impaired, which disintegrates rough ER seen in C53-knockout Paneth cells. Similar phenotypes have also been seen in the liver of the C53 knockout mouse. Both systemic and liver-specific C53 knockout mice have severe liver hypoplasia. These observations suggest that C53-mediated ER-phagy plays an important role in the maintenance of mammalian ER homeostasis and development [130].

#### 5.5.4. LNPK and a New Recessive Neurodevelopmental Syndrome

A study found that the mutation of human LNPK could cause a new kind of recessive neurodevelopmental syndrome. In the gene of LNPK, two mutations have been identified: c.726delA, c.751C>T, which result in the truncation of LNPK. The patients carrying mutated LNPK have a wide range of symptoms, such as hypotonia, epilepsy, mental retardation, and hypoplasia of the corpus callosum [131]. It is worth investigating whether LNPK contributes to the pathogenesis through ER-phagy.

## 6. Concluding Remarks

ER-phagy is highly conserved from yeast to mammals. The orthologs of yeast ER-phagy players can be also found in humans, for example, Atg40/FAM134B, Epr1/CALCOCO1, and Lst1/SEC24C. Studies from different model organisms have greatly expanded our understanding of ER-phagy. However, there are still a lot of unknowns about this important ER-quality control pathway. For instance: How do the ER-phagy receptors recognize the substrate specifically in response to different stimulus? How do ER-phagy receptors recruit other autophagic machinery to regulate the initiation, extension, and closure of the autophagosome? How is the recognition of substrates by ER-phagy receptors coupled with the formation of the autophagosome? How are ER-phagy receptors regulated by upstream signals? How the integrity of ER is maintained after the subdomain is severed from the contiguous network? What are the physiological function and pathological significance of ER-phagy in specific tissues and organs?

Besides, another central question is the relationship between ER-phagy and other selective autophagy. Considering that cells face various stimuli from inside and outside all the time, the likely answer is that they work coordinately. To maintain cellular homeostasis efficiently, cells respond to different stimuli by adopting different kinds of selective autophagy. However, it is worth noting that the relationship between stimulus and selective autophagy pathway is not corresponding. The same stimulus can trigger different types of selective autophagy, and one type of selective autophagy can also respond to multiple stimuli. A common factor of inducing selective autophagy is nutrition insufficiency [132]. Different organelles respond to different types of nutrient deficiencies. For example, in the absence of amino acids or nitrogen, ER, proteasomes, and ribosomes are induced to degrade through selective autophagy pathways [133,134,135]. While carbon resources are shifted, mitophagy or pexophagy can be induced [35,136]. Selective autophagy also responds to specific stimuli. Ferritinophagy degrades ferritin when the iron ions are unavailable; xenophagy is induced when pathogens invade; aggrephagy is increased when protein aggregates accumulate [136]. Interestingly, besides nutrition insufficiency and structure damage, ER-phagy can also be triggered by misfolded or aggregation-prone proteins [115], UPR [16], ribosome stalling [69] and viral infection (see Section 5.4). Thus, the relationship between ER-phagy and other selective autophagy is complex. Although ER-phagy is mostly used by cells to control the quality of protein synthesis and maintain ER homeostasis, it could be involved in other biological processes such as pathogen invasion together with other selective autophagy pathways. Furthermore, a recent study found that mitochondrial oxidative phosphorylation (OXPHOS) can promote ER-phagy independent of AMPK [137]. Inhibiting OXPHOS suppresses ER-phagy but enhances general autophagy [137]. These findings suggest that other organelles such as mitochondria, Golgi, or late endosomes might crosstalk with ER-phagy.

Moreover, comprehensive studies are required to address how ER-phagy crosstalk with other ER-quality maintaining pathways. It has been found that cells use various pathways, including ER-phagy, ERLAD, and ERAD to clear misfolded proteins or large-molecular-weight protein aggregates [15,20,115,116]. However, it remains largely elusive how these different disposal pathways are coordinated. A possible working model is that these pathways recognize misfolded peptides/proteins at different stages and dispose of them. For example, cells use three means to control the quality of procollagen synthesis. When mutated single chains of procollagen are produced, cells use ERAD to clear them [112]. If these mutated single chains form trimers or misfolded proteins, they will be ubiquitinated and degraded by micro-ER-phagy [23]. When misfolded procollagen become larger aggregates, cells employ macro-ER-phagy meditated by FAM134B-CALNEXIN complex to remove them [40]. The same principle may apply to ATZ [15,20], NPC1 I1076T mutation [90] and *Akita* proinsulin [115].

In addition, the interplay among different ER-phagy receptors is still puzzling. Studies have found that TEX264, FAM134B, and CCPG1 work collectively but independently to mediate ER-phagy under nutrient starvation [17,26]. A genetic study indicates that ATL2 might act downstream of FAM134B [39]. RTN3L can restore the ER-phagy defect in ATL3 deficient COS7 cells [27]. Also, it is speculated that ER-phagy receptors such as SEC62, CCPG1, and C53 may coordinate with each other as they all respond to UPR [16,28,69]. Although some research reveals the relationship of different ER-phagy receptors, a comprehensive study is still needed to systematically explore the tissue- and context-specific interactions among these known and yet-to-be-identified unknown receptors.

Many associations between ER-phagy and human diseases have already been reported. The development of drugs and therapeutic methods by targeting ER-phagy to treat related diseases is an emerging research field at present. For example, it has been reported that loperamide can induce the expression of Cyclic amp-dependent transcription factor ATF-4 (ATF4) in glioblastoma cells [138]. With the transcriptional activation by ATF4, FAM134B, and TEX264 expression is enhanced [138]. Subsequently, excessive ER-phagy is induced and autophagic cell death of cancer cells is triggered [138]. Another drug, Z36, may also induce excessive ER-phagy and Hela cell death by up-regulating the expression of FAM134B [102]. What is more, synthetic silica nanoparticles (SNPs), with good biocompatibility and low toxicity, could accumulate in the ER of HCT116 cells and may induce ER-phagy [139,140]. These studies highlight the possibility of targeting the ER-phagy pathway in cancer cells. If the nanoparticle carriers are combined with targeted therapy drugs, they can be delivered more precisely to specific organelle of tumor cells, such as ER. When tumor cells use ER-phagy to clean up the accumulated nanoparticle in the ER, on the one hand, targeted therapeutic drugs can be released; and on the other hand, excessive ER-phagy could promote autophagic death of tumor cells [140]. Nevertheless, some progress has been made about drugs targeting ER-phagy or autophagy, these drug candidates are still at a very early stage of development [141].

In conclusion, although a lot is known about ER-phagy and its role in physiological and pathological conditions, there are still lots of unknowns to explore. In a similar line to other kinds of selective autophagy, such as xenophagy and mitophagy, efforts to uncover the mechanism of ER-phagy will benefit human health greatly.

## Data Availability

No new data were created or analyzed in this study. Data sharing is not applicable to this article.
